# A meta-analysis of visual orienting in autism

**DOI:** 10.3389/fnhum.2013.00833

**Published:** 2013-12-09

**Authors:** Oriane Landry, Ashton Parker

**Affiliations:** Psychology Department, Dalhousie UniversityHalifax, NS, Canada

**Keywords:** visual orienting, Posner task, autism, meta-analysis, attention

## Abstract

**Background**: Visual orienting is inconsistently reported to be impaired in autism.

**Methods**: We conducted a meta-analysis on visual orienting in autism. We focused on studies that used a Posner-type task. A total of 18 research papers published between 1993 and 2011 were included in our meta-analysis. We examined the effects of differences in experimental design as well as differences in participant samples. We examined both orienting reaction times of participants with autism, and the effect size relative to comparison group in each experiment.

**Results**: We found that participants with autism oriented across conditions (mean orienting effect = 40.73 ms), which was of an overall smaller magnitude than that of comparison groups (Cohen's *d* = 0.44). Participants with autism were most impaired on arrow cue tasks, and least impaired on eye-gaze cue tasks, more impaired with rapid trials, and the impairment increased with age.

**Conclusions**: Variations in experimental design and participant age group contribute to whether participants with autism appear impaired at visual orienting. Critical gaps exist in the literature; developmental studies are needed across and comparing broader age ranges, and more attention should be focused on basic endogenous orienting processes.

## Introduction

In the classroom, as in the world, children can only learn about that which they attend. Selecting where to attend in the world is termed orienting. While the skill of visual orienting in autism has been of interest to researchers for the past 20 years there is no consensus in the literature as to how, if at all, visual orienting differs in autism. Clarifying the research on visual orienting will improve our understanding of the neurodevelopmental trajectory of autism. The single most widely used task for measuring visual orienting is the Posner task (Posner, 1980). Different variants of this task have been used by investigators to study visual orienting in autism, arriving at often contradictory results. Our goal was to analyze the published corpus of papers that have used a variant of the Posner task in persons with autism in an attempt to clarify the murky question about the nature of visual orienting in autism.

In a typical Posner task, participants are instructed to detect, localize, or identify a target when the target appears. Targets are preceded by cues that validly, invalidly, or neutrally prime a target's location. Participants are faster to respond to a target in a validly cued location than an invalidly cued location because attentional resources are directed to the cued location in advance of the target appearance. The reaction time advantage of valid over invalid is referred to as the *orienting effect*. In studies employing a neutral condition, the advantage of a valid over a neutral cue is referred to as the *benefit* of the valid cue and the disadvantage of an invalid relative to a neutral cue is referred to as the *cost* of the invalid cue.

Visual orienting is often classified in two ways: exogenous and endogenous, although there has been considerable debate recently about this distinction with respect to the formerly synonymous distinction of automatic and voluntary orienting (e.g., Enns and Trick, 2006; Ristic and Kingstone, 2012). We will operationally define exogenous and endogenous in the following manner: exogenous orienting occurs in response to an external stimulus, which causes an individual's attention to be drawn toward the location of that stimulus—the cue and cued locations are the same. An oft-cited example is directing attention toward a flash of lightening. Endogenous orienting, on the other hand, occurs in response to some kind of symbolic cue (or indicator) directing attention to a specific location but away from the cue—the cue and cued locations are not the same. Arrows, pointing gestures, and directional eye-gaze are examples of endogenous cues. Attention is directed away from these cues toward the direction that they specify. Endogenous orienting is often considered as being more ‘goal-driven’ than exogenous orienting. Namely, an individual's goals and motivations will have a greater impact in the way they redirect attention during endogenous relative to exogenous orienting tasks. Both types of orienting have been studied with individuals with autism.

Early studies reported deficits in exogenous orienting in autism (Casey et al., 1993; Harris et al., 1999) leading to conclusions that orienting as a whole is impaired in autism. These conclusions were challenged on the basis that these earlier studies had poorly matched comparison groups. A later study reported that exogenous orienting was intact when the developmental level of participants was taken into account (Iarocci and Burack, 2004). Pruett et al. (2011) reported likewise that children with autism showed remarkably similar patterns to typically developing children on exogenous and endogenous orienting, including to peripheral, arrow, and eye-gaze cues conditions, and with high proportions of valid trials, rendering the cue predictive of target locations, or equal proportions of valid and invalid trials, rendering the cue non-predictive. The only condition on which children with autism differed was predictive peripheral cues. In short, more recent studies of exogenous visual orienting in autism, using more appropriate comparison groups, have concluded that exogenous orienting is not as impaired as previously, thought.

Likewise, early research on endogenous orienting in autism was mixed. On the one hand, Wainwright-Sharp and Bryson (1993) reported that adults with autism did not orient in response to rapidly presented central arrow cues, although they did orient for longer cues. In contrast, several other studies reported orienting patterns in autism that were either similar to comparison participants (Kuhn et al., 2010; Greene et al., 2011) or were unusual in terms of laterality (Vlamings et al., 2005) as well as in time-course and magnitude (Senju et al., 2004; Landry et al., 2009). Various attempts have been made to explain the underlying mechanisms. Burack et al. (1997) suggested that interpreting the symbolism of the cue, not orienting *per se*, is the challenging aspect of the task for individuals with autism. Landry et al. (2009) postulated a temporal explanation specific to acting upon symbolic information independent of reflexive responses to exogenous cues. Explaining mechanisms, however, requires a more complete understanding of the behavioral deficits. Critically, a complete understanding of endogenous orienting to non-social cues is needed to understand endogenous orienting to social cues, just as a complete understanding of exogenous orienting provides context for understanding endogenous, orienting.

These inconsistencies are compounded by the heterogeneity of both research designs and participant characteristics employed in the research. These issues need to be resolved in order to contextualize orienting in the neurodevelopmental course of autism. Thus, to better characterize the nature of the deficits in visual orienting in autism, we carried out an exploratory meta-analysis to examine the effects that a number of variables might have on visual orienting performance in individuals with autism; these variables include cue type (e.g., gaze, arrow, or peripheral cue), contingency (predictive vs. non-predictive designs), aspects of task timing, complexity of response demands, and demographic variables such as age and IQ. Specifically, our goal was to examine the effects of these variables on both measured orienting within the autism samples, as well as the degree to which autism samples differed from comparison groups. We restricted our search to Posner type tasks as these are the most frequently employed tasks used to measure visual orienting in populations with autism. While theoretically informative, related tasks such as the Gap-Overlap (e.g., Landry and Bryson, 2004; Elsabbagh et al., 2009), a non-cued task measuring the temporal properties of disengagement in orienting, and the Attention Network Test (e.g., Keehn et al., 2010), a more complex task that combines orienting with other aspects of attention, have been used in a very limited number of studies with participants with autism and thus direct comparison would not be, appropriate.

## Method

### Sample of studies

A literature search was conducted using Pubmed and search terms “visual orienting” OR “exogenous orienting” OR “attention cuing” OR “attention cueing” OR “Posner task” AND autism” for articles published prior to March 2011, resulting in 125 articles. Of these, 90 were excluded as they were not experiments containing a Posner type task, and 14 were excluded as they did not include at least one participant group diagnosed with autism or autism spectrum disorders. Two studies were excluded as no reaction times were reported (Rinehart et al., 2002; Renner et al., 2006). One additional study was excluded as the experimental task examined orienting in several modalities simultaneously (Courchesne et al., 1994). Eighteen research papers met criteria, reporting a total of 21 experiments. Three experiments included saccades as the only dependent measure of reaction time; these were not included in the overall analyses, but are described for comparison.

### Moderator variables

We recorded demographic and experimental design data from each of the studies to serve as potential moderator variables. These variables are summarized in Table 1.

**Table 1:** **Demographic and experimental design data collected from target studies**.

**DEMOGRAPHIC DATA**	
Participant ages	Mean and standard deviation of the autism sample
Number of participants	Number of participants with autism and number of comparison participants
Sex	Number of males and females included
Mental age and IQ	Mean, standard deviation, and range, of all IQ measures and/or mental age equivalents reported in the study, as well as IQ test name
Information on comparison group	Age, IQ, mental age, and sex of comparison participants (mean, standard deviation, and/or range)
**DESIGN ASPECTS**	
Alerting tone	Yes or no
Cue	Described
Stimulus onset asynchrony (SOA)	All SOAs (ms) included in the study; SOA is the elapsed time from cue onset to target onset
Fixation point	Yes or no
Pre-cue stimulus	Yes or no. If yes, described
Neutral condition	Yes or no
Target stimulus	Described
Inter trial interval (ITI)	ITI in ms, and whether feedback was given during this ITI
Overlap	Was there temporal overlap between the cue offset and target onset, yes or no
Type of response	Detection, localization, or identification

### Dependent measures

We recorded mean reaction times and standard deviations for each condition [valid, invalid, neutral, by Stimulus Onset Asynchrony (SOA) and task]. Where raw reaction times and standard deviations /error were not reported in the manuscript, study authors were contacted if the study was recent; otherwise data was estimated from graphs (2 studies). One study was dropped (Ristic et al., 2005) because estimates could not be made from the graphs. Two dependent measures were extracted from the data to be analyzed:

- Reaction time measure of the magnitude of the orienting effect (invalid RT- valid RT) for participants with autism. The magnitude of the orienting effect provides us with a descriptive measure of orienting performance in autism independent of comparison groups. This allows us to examine which variables influence orienting within autism, without judgments relative to a comparison group. This measure addresses the question *do individuals with autism orient?*
- Cohen's *d* effect sizes, a standardized measure of the difference between group means. Cohen's *d* effect sizes (autism vs. comparison group) were calculated separately for invalid and valid RTs. Cohen's *d* effect sizes provide us with an examination of whether orienting is *intact* or *impaired* relative to comparison groups across different types of task, SOA, and age of participants, or whether there are baseline reaction time differences between groups. This measure addresses the question *are individuals with autism impaired at orienting?*

All data were analyzed using R (R Development Core Team, 2012) and the R packages *lme4* (Bates et al., 2012) and *languageR* (Baayen, 2007). Each dependent measure was analyzed using linear mixed effects models (LME), an extension of linear regression that allows the specification of nested random effects. This method was chosen to control for the effect of “Study”. Analyses were weighted by the sample size of each study. Normality and homogeneity were checked by visual inspection of plots of residuals against fitted values. Models were compared using likelihood ratio tests, and MCMC-estimated *p*-values are presented throughout.

## Results

Demographic data was sufficient to include mean age as a variable, although the range included in some studies was so large that this should be interpreted cautiously. Ten included children 7–12 years old, four included adolescents and four included adults. Reported measures of IQ were so diverse that they could not be meaningfully included in the analysis. We classified whether the comparison group was well-matched for developmental level with the autism group, whether by reported mental age or by combined age and IQ. We use the inclusive term of comparison group, however, in no study was the comparison group explicitly identified as anything other than typically developing. Nine experiments were judged as having reasonably well matched comparison groups (50%), six were judged as unknown because information was missing or the range of IQs/mental ages in the autism group extended substantially lower than the comparison group, and three were judged as poorly matched in that the IQs/mental ages of the groups differed substantially (although one of these used IQ as a covariate in analyses; we used the covariate estimated means in our analyses). This data is presented in Table 2.

**Table 2:** **Age and IQ details of participants with autism and comparison groups**.

**Study**	***n***	**# Male participants**	**Age mean (*SD*) or ± *SE* as reported**	**Age range**	**IQ and/or mental age**		**Are groups well matched?**
	**AUTISM GROUP**						
Casey et al., 1993	10	all	29.2 (8.6)	19–41	WAIS full scale IQ 82(13), 65–107		No—Substantial IQ difference (Adults, Age matched only)
deJong et al., 2008	30	24	10.7 ± 1.8		Dutch version of WISC full 108.4 ± 2.6; verbal 113.3 ± 2.7; perf 101.4 ± 3.1		Yes—Age and IQ (HFA)
Goldberg et al., 2008	22	16	10.47 (1.77)	8–13	WISC full 100.6 (15.54)		No—IQ difference, but used as covariate
Greene et al., 2011	22	20	12.95 (2.46)	9–17	WASI or WISC full 103.25 (13.93)		Yes—Age and IQ (HFA)
Harris et al., 1999 Autism group*	7	all	7.82 (1.7)		PPVT 46.6 (11.1)	IQ 87.7 (12.3)	No—Substantial IQ difference (Children, Age matched only)
Harris et al., 1999 PDDNOS group*	5	4	4.21 (0.8)		PPVT 72.0 (18.9)	IQ 105.4 (13.7)	Unknown—small but FSIQ isn’t as badly matched
Iarocci and Burack, 2004	14	11	11.6 (4.9)		K-BIT mental age 7.2 (0.99)		Yes—Mental age matched
Kylliainen and Hietanen, 2004	12	11	9;11 (1;10)	7;4–14;1	WISC-R FS 91(17), perf 95(16), verbal 90(19); MA 9;3 (2;11), 6;8–16;0		Yes—Mental age matched
Landry et al., 2009	18	na	11.52(3.07)		perf.(WASI)—99.50(15.53) WASI blocks—29.39(20.70) WASI matricies—21.22(7.11); PMA—11.51(3.74)		Yes—Mental age matched
Pruett et al., 2011	27	22	11.1 (1.2)	9–12	WISC scaled blocks 12.3 (2.8) scaled vocab 10.3 (2.6)		Yes—Age and IQ (HFA)
Rutherford and Krysko, 2008	23	22	25.9 (9.6)	18–52	WAIS full 100.1 (15.0) 76–145; verbal 102.6 (14.8) 77–144; perf 96.9 (16.0) 74–136		Yes—Age and IQ (HFA)
Senju et al., 2004—Experiment 1	11	8	10.11	9.7–12.6			Unknown—CA matched and no IQs; presumed to be normal range based on educational placement
Senju et al., 2004—Experiment 2	26	23	9.6	7.6–12.3			Unknown—CA matched and no IQs; presumed to be normal range based on educational placement
Swettenham et al., 2003—Experiment 1	15	na	10;2 (0;9)	8;8–11;2	Raven’s progressive matrices raw 37.6 (10.3)		Yes—Age and IQ (HFA)
Swettenham et al., 2003—Experiment 2	15	na	10;2 (0;9)	8;8–11;2	Raven’s progressive matrices raw 37.6 (10.3)		Yes—Age and IQ (HFA)
Uono et al., 2009	11	8	17.5 ± 6.5	9–30	Japanese versions of WAIS or WISC full = 107.73 (9.05); viq 107.55 (13.06); piq 104.55 (10.43)		Unknown—Comparison group contains more restricted age range, no children, and no IQ measures (although normal range is assumed, not indicated if they are undergraduates or community sample)
Vlamings et al., 2005	19	16	22.53 (4.96)				Unknown—CA matched and IQs not reported (only reported to be “in normal range” as per selection criteria)
Wainwright-Sharp and Bryson, 1993*	11	all	20.4	13–27	Raven’s progressive matrices standard score 5–95; PPVT Standard Score 89, 64-122		Unknown—range of scores on standardized tests extends much lower in ASD group
	**TYPICALLY DEVELOPING COMPARISON GROUP**					
Casey et al., 1993	10	all	29.6 (5.2)	22–35	124 (16), 97–148 WAIS-R subtests		
deJong et al., 2008	30	24	10.6 ± 1.6		WISC full 111.5 ± 2.2; verbal 116.3 ± 2.5; perf 100.6 ± 2.5		
Goldberg et al., 2008	49	24	10.41 (1.42)	8–13	113.53 (14.59)* sig diff!!		
Greene et al., 2011	21	19	13.19 (2.44)	10–17	full 110.48 (14.10)		
Harris et al., 1999 Autism group*	15	14	7.44 (0.9)		IQ 115 (8.3)		
Harris et al., 1999 PDDNOS group*	15	14	7.44 (0.9)		IQ 115 (8.3)		
Iarocci and Burack, 2004	14	9	5.7 (0.64)		K-Bit mental age 6.4 (0.29)		
Kylliainen and Hietanen, 2004	12	11	8;11 (2;10)	6;1–16;0	WISC-R FS 106 (7), perf 102 (7), verbal 109 (8); mental age 9;5 (2;10), 6;6–16;0		
Landry et al., 2009	16	na	11.00 (2.66)		WASI—114.44 (13.69) WASI blocks—38.87 (17.85) WASI matricies—24.07 (4.92); PMA—12.49 (3.74)		
Pruett et al., 2011	25	20	11 (1.2)	9–12	WISC scaled block 11.8 (2.5) scaled vocab 11.2 (2.1)		
Rutherford and Krysko, 2008	23	22	26.5 (9.5)	18–53	WAIS full 104.4 (13.4) 77–135; verbal 104.4 (11.4) 79–125; perf 103.7 (16.0) 75–138		
Senju et al., 2004—Experiment 1	14	6	11.1	10.0–12.2			
Senju et al., 2004—Experiment 2	38	25		7.7–12.5			
Swettenham et al., 2003—Experiment 1	15	na	10;2 (0;9)	8;8–11;2	Raven’s progressive matrices 37.7 (10.4)			
Swettenham et al., 2003—Experiment 2	15	na	10;2 (0;9)	8;8–11;2	Raven’s progressive matrices 37.7 (10.4)			
Uono et al., 2009	11	8	19.5 ± 2.2	18–26			
Vlamings et al., 2005	19	all	23.05 (3.70)				
Wainwright-Sharp and Bryson, 1993*	11	all	20.6	14–27	Raven’s progressive matrices standard score 90–99; PPVT 117, 97–133 (std)			

^*^not included in effect size analysis (missing data).

The variety of designs employed are shown in Table 3. Two experiments used alerting tones, 15 included fixation points, and five included a pre-cue stimulus. Four experiments included exogenous cues, seven included arrow cues, and eight included eye-gaze cues. Four experiments included predictive cues (ranging from 67–80% valid cues), 13 included non-predictive cues, and one included counter-predictive cues. Six studies included neutral conditions. The SOAs ranged from 100–1100 ms, and in nine experiments the cue and target overlapped temporally. In only one experiment did participants have to identify the target and filter competing distracter symbols, rather than simply localize or detect. Five studies did not report inter-trial interval lengths.

**Table 3:** **Designs employed in orienting tasks**.

**Study**	**Alerting tone**	**Fixation point**	**Pre-cue stimulus**	**Cue**	**Predictive?**	**Neutral condition**	**SOA**	**Target stimulus**	**Non-target stimulus**	**Overlap (cue and target at same time)**
Casey et al., 1993	No	Yes, central plus sign, with 2 boxes on either side (5 s)	No	exogenous	Yes, valid 80%	Yes, either both got inner boxes or neither	100 ms, 800 ms	* in center of box L or R	No	Yes
deJong et al., 2008	No	Yes, fixation dot (1100–1700 ms)	Yes	Gaze cue	No, valid 50%	No	813 ms	black dot to L or R	No	Yes
Goldberg et al., 2008	No	Yes, fixation cross (750 ms)	No	Gaze cue	No, valid 50%	No	200 ms, 700 ms	* to L or R	No	No
Greene et al., 2011	No	Yes, central cross (700 ms)	No	Arrow and gaze cues	No, valid 50%	Yes, doubly inverted arrow or look straight	300 ms	X' to L or R	No	Yes
Harris et al., 1999	No	Yes, central white cross. With green boxes on L and R	No	Exogenous	Yes, valid 67%	Yes, both boxes brighten (1/6)	200 ms, 1000 ms	* to L or R	No	Yes
Iarocci and Burack, 2004	Yes	No	No	Exogenous	No, valid 50%	Yes (center)	150 ms	O/+ to R, L, or midpoint	Yes, 4 distractor symbols (50%)	No
Kylliainen and Hietanen, 2004	No	Yes, central cross (1000 ms)	No	Gaze cue	No, valid 50%	Yes, look striaght	200 ms, 800 ms	* to L or R	No	No
Landry et al., 2009	No	No	No	Arrow cue	No, valid 50%	No	200 ms, 400 ms, 700 ms, or 1100 ms	X' to L or R	No	No
Pruett et al., 2011	No	Yes, fixation cross, neutral arrow or face (1, 1.5, or 2s)	No	Exogenous, arrow, and gaze cues	50% and 80% valid	No	150 ms, 800 ms	* to L or R	No	Yes
Rutherford and Krysko, 2008	No	Yes, fixation point (1s or 2s)	No	Gaze cue	No, valid 50%	No	100 ms, 800 ms	white astrix on photo to R or L	No	Yes
Senju et al., 2004—Experiment 1	No	Yes, central cross (675 ms)	Yes, eyes-closed face or square—900 ms	Arrow and gaze cues	No, valid 50%	No	100 ms, 300 ms, 700 ms, or 1,000 ms	* to L or R	No	No
Senju et al., 2004—Experiment 2	No	Yes, central cross (675 ms)	Yes, eyes-closed face or square—900 ms	Arrow and gaze cues	Counter (valid 20%)	No	100 ms, 300 ms, 700 ms	* to L or R	No	No
Swettenham et al., 2003—Experiment 1	No	Yes, central cross 1 or 2s	Yes, eyes forward 500 ms	Gaze cue	No, valid 50%	No	100 ms, 800 ms	* to L or R	No	Yes
Swettenham et al., 2003—Experiment 2	No	Yes, central cross 1 or 2 s	Yes, eyes forward 500 ms (inverted)	Gaze cue	No, valid 50%	No	100 ms, 800 ms	* to L or R	No	Yes
Uono et al., 2009	No	Yes, fixation cross (600 ms)	No	Gaze cue	No, valid 50%	No	460 ms	T' to L or R	No	Yes
Vlamings et al., 2005	No	Yes, neutral arrow or gaze (500 ms)	No	Arrow and gaze cues	No, valid 50%	No	1500 ms	A' to L or R	No	No
Wainwright-Sharp and Bryson, 1993	Yes	Yes, central, asteirx 1–2 s	No	Arrow cue	Yes, valid 80%	Yes, line	100 ms, 800 ms	Cross to L or R	No	No

### Magnitude of the orienting effect in autism across studies

**Figure 1:**
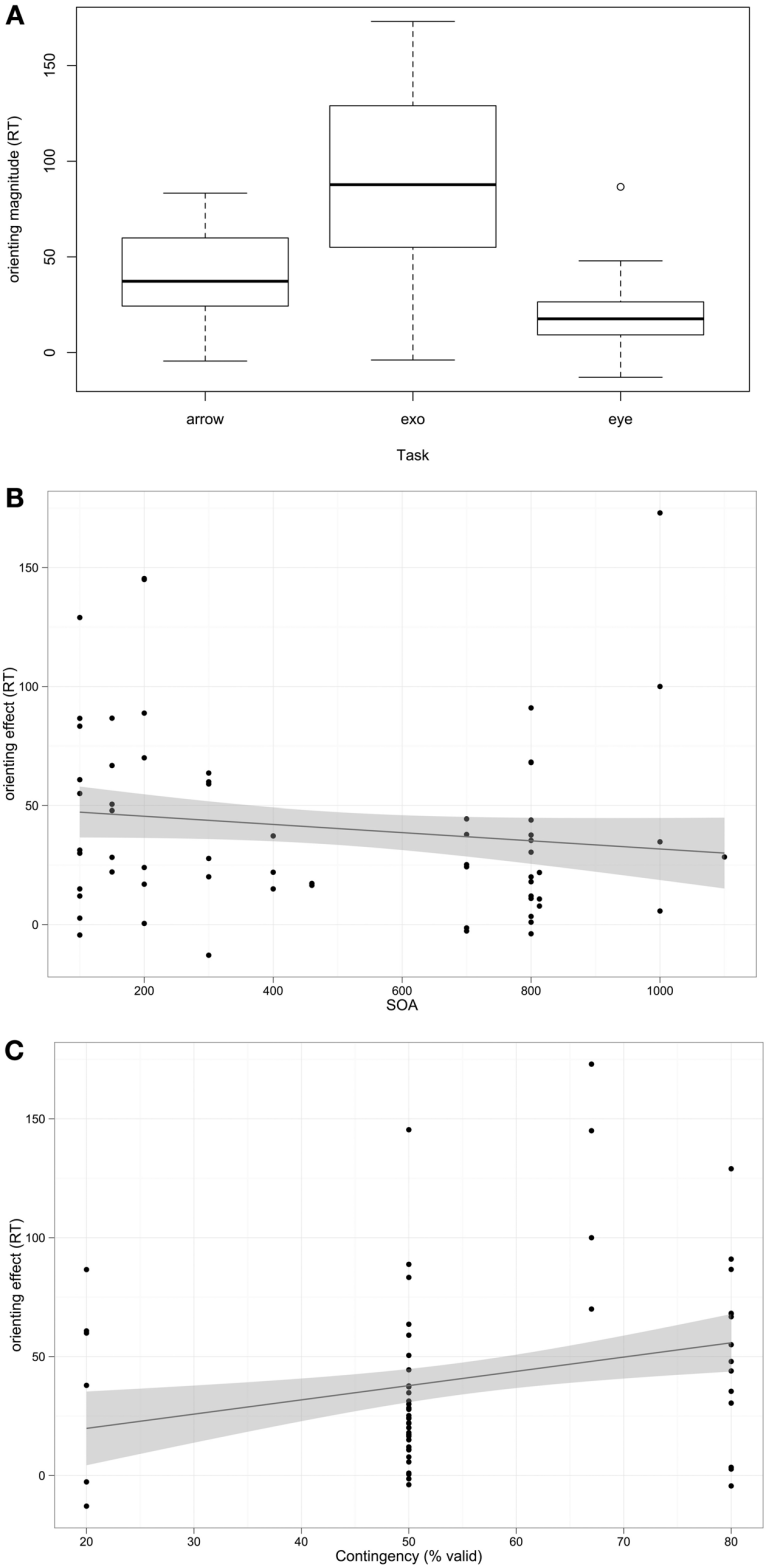
**Associations between predictors and orienting magnitude RT among participants with autism**. **(A)** Box-plot of orienting effect across task types. **(B,C)** Scatterplots with linear regression line of best fit.

Overall, the mean RT orienting effect for participants with ASD was 40.73 ms (95% C.I 33.82–47.64); as this is significantly greater than 0 (*t*_(125)_ = 11.67, *p* < 0.001), the general finding across studies is that participants with ASD orient. The next question was which factors influence orienting performance. Fixed effects (predictors) included in the model were *Task* type (exogenous, arrow, or eye-gaze cue), *SOA*, *Contingency*, whether the cue and target temporally *Overlap*, and mean *Age* of participant sample. The random effect included in the model was *Study*. The analysis was weighted by the sample size of each study. The best fitting model included Task, SOA, and Contingency (log likelihood ratio = −768.93). Orienting RT differed as a function of Task; orienting RT magnitude was weaker in Eye Gaze than Arrow cuing (β = −18.47, *p* = 0.001), but there was no significant difference between Exogenous and Arrow cuing (β = −1.34, *p* = 0.58, *ns*). SOA was negatively associated with orienting RT (β = −0.03, *p* = 0.001). Contingency contributed significantly to the model but the positive association with orienting RT was only a trend (β = 0.67, *p* = 0.08). Age was not associated with orienting RT, nor was the categorical distinction of whether the cue and target overlapped temporally during the task; models including these predictors did not significantly improve model fit. These associations are depicted in Figure 1.

### Effect sizes relative to comparison groups

**Figure 2:**
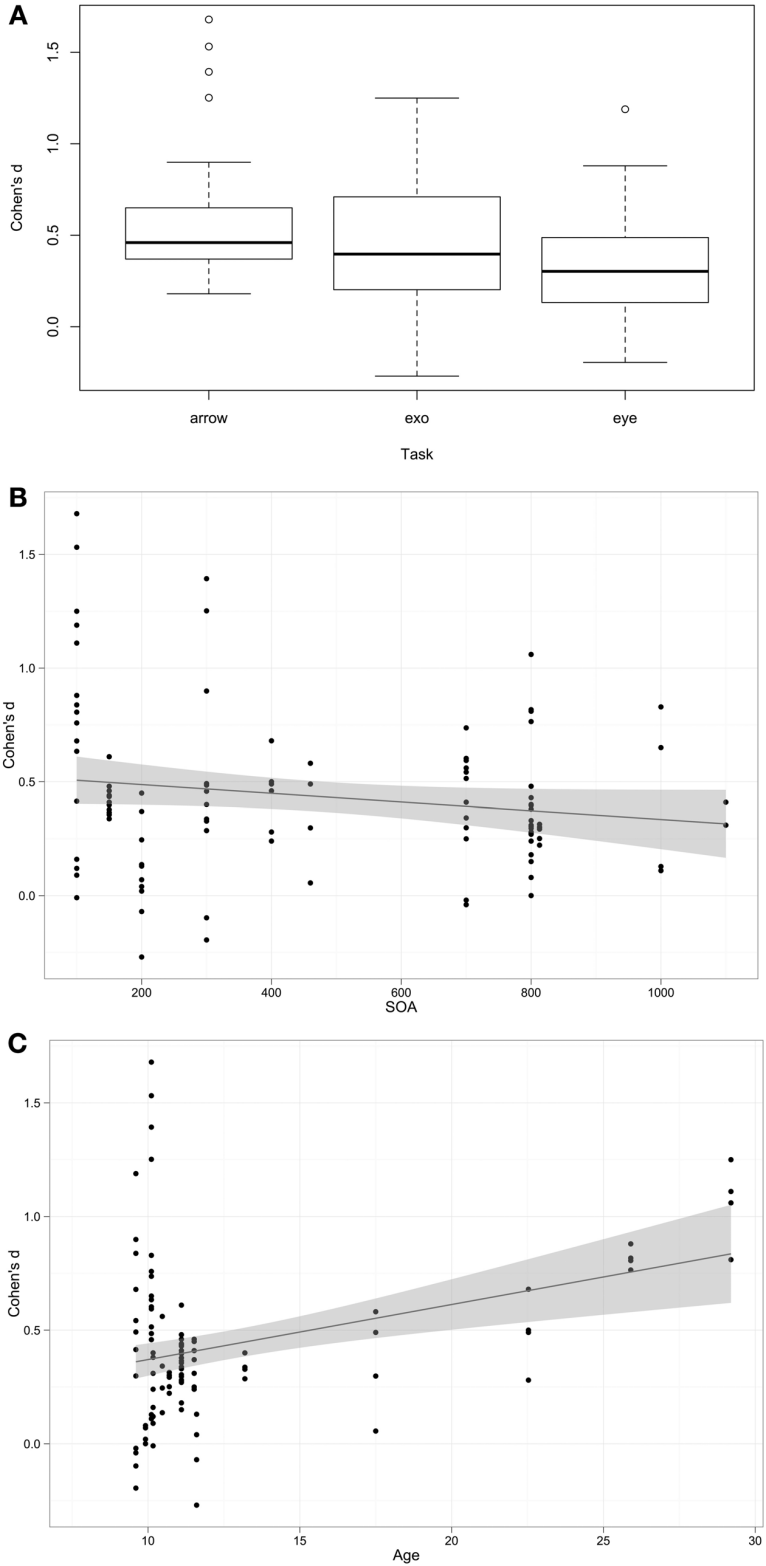
**Association between predictors and Cohen's d effect size**. **(A)** Box-plot of Cohen's *d*-values across task types. **(B,C)** Scatterplots with linear regression line of best fit.

Cohen's *d* is a standardized measure of the difference between participants with autism and their comparison groups. As such, larger values are indicative of larger autism impairments. For the purposes of interpretation, Cohen's *d* > 0.8 is considered to be a large effect, >0.5 is a medium effect, and >0.2 is a small effect. Overall a mean Cohen's *d*-value of 0.44 (95% C.I. 0.37–0.50) was found, indicating that overall a small autism impairment was observed as the effect size was significantly greater than 0, *t*_(105)_ = 12.90, *p* < 0.001. The next question was what factors influence impairment. Fixed effects (predictors) included in the models were *Cue* (valid or invalid), *Task* type (exogenous, arrow, or eye-gaze cue), *SOA*, *Contingency*, mean *Age* of participant sample, whether there was cue-target *Overlap* (Y/N), and whether groups were well *Matched* (Y/N/Unknown). The random effect included in the model was *Study*. The analysis was weighted by the sample size of each study. The best fitting model included *Task, SOA*, and *Age*, log likelihood ratio = −176.3. Participants with autism were more impaired on Arrow than Eye Gaze (β = −0.22, *p* < 0.001) conditions, with no significant difference between Arrow and Exogenous conditions (*p* = 0.22), impairment increased with age (β = 0.03, *p* = 0.016), and decreased as SOA increased (β = −0.0002, *p* = 0.015). These associations are shown in Figure 2. Models including *cue*, *contingency*, *overlap*, and *matched* did not improve model fit.

## Discussion

We conducted a meta-analysis of the research examining visual orienting in autism. We focused exclusively on Posner-type visual orienting experiments as these are the most frequently used tasks permitting direct comparison across studies. We examined two dependent measures. The first dependent measure was orienting reaction times, to assess whether individuals with autism orient and what conditions influence the magnitude of orienting. The second dependent measure was Cohen's *d* effect sizes, a standardized measure of the differences between groups, thus providing a metric of impairment. We concluded that overall, participants with autism orient, and this orienting is impaired relative to comparison participants. The average Cohen's *d* effect size across studies was 0.44, a small effect.

In considering the question of whether orienting is impaired in autism, we also have to consider the multitude of factors that may influence orienting, both in terms of differences in experimental design as well as differences in participant samples. We found that individuals with autism were most impaired on arrow cuing tasks and least impaired on eye-gaze cuing tasks, were more impaired at shorter SOAs, and that relative impairment increased with age. Nevertheless, even under the most favorable conditions, participants with autism were impaired, and the effect size was small. There is not enough data to examine which combinations of favorable conditions might eliminate the autism disadvantage, although one could speculate based on the experiments in which effect sizes are less than *d* = 0.2, summarized in Table 4; little to no autism impairment was found in studies that all included younger participants, in non-predictive exogenous or eye-gaze conditions.

**Table 4:** **Magnitude of the orienting effects (invalid—valid RT) and overall Cohen's d effect sizes for each experiment, presented in descending order from largest autism impairment to largest autism advantage**.

**Study**	**Cue**	**Autism orienting effect (RT)**	**Comparison orienting effect (RT)**	**Cohen's d**
Casey et al., 1993	Invalid	85.75	26.50	1.16
Casey et al., 1993	Valid	85.75	26.50	0.96
Senju et al., 2004—Experiment 1	Valid	35.46	6.65	0.82
Rutherford and Krysko, 2008^a^	Valid	13.50	5.00	0.82
Rutherford and Krysko, 2008^a^	Invalid	13.50	5.00	0.81
Senju et al., 2004—Experiment 1	Invalid	35.46	6.65	0.72
Senju et al., 2004—Experiment 2	Invalid	38.27	‒8.90	0.54
Kuhn et al., 2010^b^	Valid	18.18	19.57	0.54
Kuhn et al., 2010^b^	Invalid	18.18	19.57	0.51
Vlamings et al., 2005	Invalid	18.50	18.50	0.50
Vlamings et al., 2005	Valid	18.50	18.50	0.48
Landry et al., 2009	Invalid	31.78	18.85	0.41
Pruett et al., 2011	Invalid	36.16	18.82	0.40
Goldberg et al., 2008	Valid	‒0.47	14.25	0.40
Uono et al., 2009	Valid	16.90	17.80	0.39
Greene et al., 2011	Invalid	45.70	40.15	0.36
Pruett et al., 2011	Valid	36.16	18.82	0.34
Uono et al., 2009	Invalid	16.90	17.80	0.32
Landry et al., 2009	Valid	31.78	18.85	0.31
Greene et al., 2011	Valid	45.70	40.15	0.31
Senju et al., 2004—Experiment 2	Valid	38.27	‒8.90	0.29
deJong et al., 2008	Invalid	13.48	11.00	0.29
deJong et al., 2008	Valid	13.48	11.00	0.27
Swettenham et al., 2003—Experiment 1	Valid	24.00	30.00	0.26
Swettenham et al., 2003—Experiment 1	Invalid	24.00	30.00	0.24
Swettenham et al., 2003—Experiment 2	Valid	11.50	26.50	0.24
Goldberg et al., 2008	Invalid	‒0.47	14.25	0.24
Swettenham et al., 2003—Experiment 2	Invalid	11.50	26.50	0.12
Chawarska et al., 2003—Experiment 2^b^	Valid	‒6.00	‒1.00	0.07
Kylliainen and Hietanen, 2004—orienting	Invalid	12.50	22.50	0.05
Kylliainen and Hietanen, 2004—orienting	Valid	12.50	22.50	0.04
Iarocci and Burack, 2004	Invalid	117.10	79.15	−0.02
Iarocci and Burack, 2004	Valid	117.10	79.15	−0.07
Chawarska et al., 2003—Experiment 2^b^	Invalid	‒6.00	‒1.00	−0.10
Chawarska et al., 2003—Experiment 1^b^	Valid	9.00	12.00	−0.76
Chawarska et al., 2003—Experiment 1^b^	Invalid	9.00	12.00	−1.18
Wainwright-Sharp and Bryson, 1993^c^	–	25.48	29.00	NA
Harris et al., 1999^c^	–	122.00	80.00	NA

*Positive values > d = 0.2 indicate autism impairment. Negative values <, d = −0.2 indicate autism advantage. Orienting effects are the RT difference between invalid and validly cued conditions. Cohen's d-values were calculated separately for invalid and validly cued conditions within each experiment*.

a eye gaze condition only.

b saccadic RT (not included in analyses).

c insufficient data to calculate effect size.

**Figure 3:**
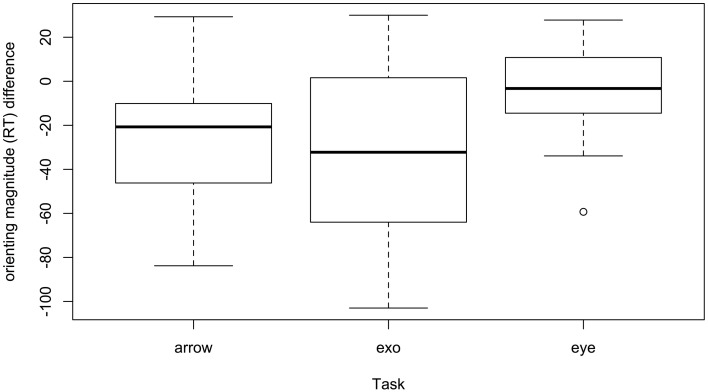
**Differences between autism and comparison participants in magnitude orienting RT**. Negative values denote larger orienting effects in autism.

Critically, participants with autism were not differentially influenced on invalid vs. valid trials (invalid mean *d* = 0.45, valid mean *d* = 0.41, *p* = 0.64), thus the impairment may simply reflect a general task impairment reflecting slower reaction times; there also was no evidence for *cue* interacting with other variables. The manner with which the data is presented in the literature does not permit calculating an effect size for the invalid–valid orienting effect itself, only for calculating effect sizes separately fo valid and invalid RTs. The vast majority of papers report valid and invalid RTs, along with standard deviations for valid and invalid RTs. From this data we were able to calculate the orienting effect (invalid - valid) but we are unable to derive a standard deviations in order to calculate the effect size for each study. For descriptive purposes we can plot the difference in orienting effects for autism and comparison samples. Figure 3 presents the differences in orienting RT (invalid - valid RT) between autism and comparison samples. Most values are negative, reflecting larger orienting RTs among participants with autism (autism mean = 40 ms; comparison mean = 20 ms). Presented as a function of task, the box-plot shows the median orienting RT difference between samples is lowest in eye-gaze tasks, with autism samples producing an orienting effect that is on average differing by less than 10 ms from that of comparison groups. In arrow tasks, autism samples differ by an average of 20 ms, and in exogenous tasks by an average of 30 ms.

Eye gaze cuing was the most frequently used in the literature, accounting for slightly more than half of the included studies, but the effect size for arrow cues was 0.2 *d* higher than for eye gaze, suggesting further research is needed on non-social endogenous cues. Impairment was also noted for exogenous cuing, however, high variability and two poorly matched experiments using this design also suggest further research is needed on this task. Only one study examined all three tasks in both predictive and non-predictive conditions, in a well-matched sample of children (mean age 11), finding group differences only on predictive exogenous cues at the shorter SOA (Pruett et al., 2011). We hypothesize that the same experiment, carried out with adolescents and adults, would show increasing group differences with age.

Contingency, while influencing orienting reaction times, did not contribute to impairment; participants with and without autism were equally influenced by the contingency of a task. The diversity in contingencies represented in the literature is less than optimal. Nearly ¾ of studies used a non-predictive contingency, and only one study used a counter-predictive contingency. The lack of evidence for an influence of contingency on impairment may reflect this imbalance. Future research should incorporate a wider range of contingencies, and examine contingency as a factor in performance. Of particular concern is the limited number of predictive endogenous cuing experiments completed by participants with autism.

There was an interesting temporal element to the autism orienting impairment; SOA was negatively associated with orienting RT, and was also negatively associated with Cohen's *d* effect sizes. In other words, individuals with autism were most impaired in the context of rapid trials. This conclusion was previously drawn and competing theories have been put forth to explain the underlying mechanisms (Burack et al., 1997; Landry and Burack, 2009; Landry et al., 2009), but further research will be needed to tease this pattern apart. This temporal impairment may have very important implications for early development and education, as slowing down the pace of a social interaction may allow the younger child with autism a greater opportunity to orient to various cues, and *keep-up* with the interaction. Thus, we might expect parents or interventionists who are better able to synchronize to the child will achieve better results. For example, Baker et al. (2010) reported that while there were no differences in maternal sensitivity between a group with emergent ASD and a group without ASD, among the ASD mother-child dyads, sensitivity was associated with greater language gains from 18–36 months. Sensitivity to pacing of interactions may be an underlying factor in this association and should be the focus of future investigation.

Interestingly, while there were no effects of age upon orienting RT, there was a significant association between age and Cohen's *d* effect size. The implications are two-fold. First, if the effect size gets larger as the participant sample gets older, the likelihood that a given study will conclude orienting is impaired in autism depends in large part on the age at which participants are tested. For example, Iarocci and Burack (2004) argued that their more appropriate matching procedures eliminated the exogenous orienting impairment reported by previous studies, however, it could also be due to their younger participant sample. Half of all studies included children in the 7–12 year old range, and these were more likely to conclude that there was no autism impairment. Why this is such a popular age group is unclear. Studies of even younger children, while not included in the analyses as they only recorded saccadic RT, are consistent with this age effect in that toddlers showed Cohen's *d* effect sizes ranging from 0.38 to −1.18, with most closer to 0 (Chawarska et al., 2003), an effect that is arguably driven by age more than the simple change from manual response to saccadic RT; Kuhn et al. (2010) measured saccadic orienting responses in adults with ASD and found a moderate effect of *d* > 0.50.

While this may appear inconsistent with the evidence from Gap-Overlap experiments that find orienting differences in infancy predict later autism diagnosis (Zwaigenbaum et al., 2005; Elsabbagh et al., 2013), these are qualitatively different tasks. The Gap-Overlap is a non-cued orienting task and the autism impairment reported is one of disengagement; infants with autism exhibit more “sticky attention” to the central stimulus when it remains onscreen overlapping with the peripheral target stimulus. Similar results are also reported for toddlers with autism (Landry and Bryson, 2004). The Posner task on the other hand is a *cued* orienting task. We found that nearly half of the experiments included in this analysis contained temporally overlapping cues and targets, and thus we might have expected that participants with autism would be more impaired when the task contained overlapping cues and targets. We did not find this to be the case, overlapping tasks elicited a mean *d* = 0.45 while non-overlapping tasks elicited a mean *d* = 0.42. Furthermore, Chawarska et al. (2010) did not find stickier attention in toddlers with autism, in fact the toddlers with autism were less sticky when the stimuli were faces and groups didn't differ when the central stimulus was a non-social non-cue. Future studies will need to explore the potential ways in which early “sticky attention” could compromise children's acquisition of cued orienting. For example, an early overgeneralized sticky attention could signal that the child with autism is not differentiating relevant and meaningful environmental cues, while the typically developing infant is separating the signal from the noise and is both attracted to and has more difficulty disengaging from important signals.

Second, it no longer seems appropriate to ask whether orienting is impaired or not in autism, if the impairment is one that builds with age. The potential impact of slowed orienting in childhood, adolescence, or adulthood needs to be further examined. This age-related change also needs to be explored in greater depth. It does not appear to be the case that orienting effect RTs necessarily change with age, but it may be that typically developing adolescents and adults evidence greater overall speed increases with age than do individuals with autism. Given the limited number of studies and variability of designs, we were unable to explore interactions among factors, however, it is imperative that future studies approach the question developmentally, testing children as young as possible on identical tasks, and including a wider age range on the saccadic-based tasks that are appropriate for the youngest children.

The results of our meta-analysis clearly show the following three general conclusions:

First, individuals with autism orient in response to the three most frequently used cues. Second, individuals with autism evidence a temporally based impairment in visual orienting that increases with age. Third, gaps in the research exist in that the vast majority of research has been conducted with participants in late childhood using non-predictive cues, especially eye-gaze cues. Orienting effects, the magnitude of reaction time advantage of valid vs. invalid cues, were small across all studies employing this method, and Cohen's *d* effect sizes were variable, ranging from no autism impairment to substantially large autism impairments measured. The disproportionate number of studies using this methodology is not surprising given the characterization of autism as a disorder of *social* communication and behavior, with gaze aversion being a stereotypic diagnostic symptom; researchers therefore hypothesize that social orienting of attention could be a pivotal skill or core deficit and research resources are disproportionately directed toward that goal. We conclude that more research needs to be conducted on participants at different ages, ideally longitudinal, and using more consistent methods to measure orienting. What was most surprising was the paucity of research using non-predictive exogenous cues and predictive arrow cues, given these are the staples of adult cognitive research on the topic of, orienting.

It is clear that visual orienting is an important area of research in autism, with group differences reported even for infants at high-risk of developing autism and predicting those that receive a later diagnosis (Zwaigenbaum et al., 2005; Elsabbagh et al., 2009, 2013). Future studies examining orienting in younger children and more comprehensively across the lifespan are needed to better understand the course of endogenous orienting to both social and non-social cues and how subtle atypicalities in endogenous orienting might influence other emerging skills.

### Conflict of interest statement

The authors declare that the research was conducted in the absence of any commercial or financial relationships that could be construed as a potential conflict of interest.
